# Delayed emergence of subdiffractionsized mutant huntingtin fibrils following inclusion body formation

**DOI:** 10.1017/S0033583515000219

**Published:** 2015-09-09

**Authors:** Steffen J. Sahl, Lana Lau, Willianne I. M. Vonk, Lucien E. Weiss, Judith Frydman, W. E. Moerner

**Affiliations:** 1 Department of Chemistry, Stanford University, Stanford, CA, USA; 2 Department of Biology, Stanford University, Stanford, CA, USA

**Keywords:** huntingtin, Huntington’s Disease, protein aggregation, amyloid, super-resolution, single-molecule imaging

## Abstract

Aberrant aggregation of improperly folded proteins is the hallmark of several human neurodegenerative disorders, including Huntington’s Disease (HD) with autosomal-dominant inheritance. In HD, expansion of the CAG-repeat-encoded polyglutamine (polyQ) stretch beyond ~40 glutamines in huntingtin (Htt) and its N-terminal fragments leads to the formation of large (up to several *μ*m) globular neuronal inclusion bodies (IBs) over time. We report direct observations of aggregating Htt exon 1 in living and fixed cells at enhanced spatial resolution by stimulated emission depletion (STED) microscopy and single-molecule super-resolution optical imaging. Fibrils of Htt exon 1 arise abundantly across the cytosolic compartment and also in neuritic processes only *after* nucleation and aggregation into a fairly advanced stage of growth of the prominent IB have taken place. Structural characterizations of fibrils by STED show a distinct length cutoff at ~1·5 *μ*m and reveal subsequent coalescence (bundling/piling). Cytosolic fibrils are observed even at late stages in the process, side-by-side with the mature IB. Htt sequestration into the IB, which in neurons has been argued to be a cell-protective phenomenon, thus appears to saturate and over-power the cellular degradation systems and leaves cells vulnerable to further aggregation producing much smaller, potentially toxic, conformational protein species of which the fibrils may be comprised. We further found that exogenous delivery of the apical domain of the chaperonin subunit CCT1 to the cells via the cell medium reduced the aggregation propensity of mutant Htt exon 1 in general, and strongly reduced the occurrence of such late-stage fibrils in particular.

## Introduction

Like several other human neurodegenerative pathologies, Huntington’s Disease (HD) (Bates *et al*. 2015; Ross & Tabrizi, 2011) is linked to aggregation of an essential protein. Details of the aggregation process of mutant (i.e. polyQ-expanded) huntingtin (Htt) and its N-terminal fragments – notably Htt exon 1 (Htt_ex1_) – have been the subject of intense study over the last 15–20 years, ever since the genetic cause, an autosomal-dominant inherited increase in the cytosine, adenine, guanine (CAG) repeat number, was uncovered two decades ago (Huntington’s Disease Collaborative Research Group, 1993). A family of at least nine to ten disorders share this feature of polyglutamine repeat expansion, which are prominent examples of the general problem of repeat instability and expansion (Orr & Zoghbi, 2007). Much akin to other neurodegenerative diseases, e.g. Lewy bodies in Parkinson’s Disease, the major histo-morphological hallmark of HD is the aberrant accumulation of the mutant protein in dense, ubiquitin-containing cytosolic and nuclear aggregates termed inclusion bodies (IBs). These IBs have been widely acknowledged as the *endpoint of aggregation*. Electron-microscopic (EM) investigations have long identified rather large aggregates (DiFiglia *et al*. 1997), often of several micrometers diameter, amounting to substantial total fractions of the neuronal Htt sequestered inside them. Exon 1 of Htt, the leading ~100 amino acids, which was long thought to be a routine protease cleavage product but whose production was recently identified as resulting from repeat-length-dependent aberrant splicing of the exon 1 *HTT* gene (Sathasivam *et al*. 2013), is the predominant component of IBs analyzed in human autopsy brains (DiFiglia *et al*. 1997). A large body of work indicates that shorter Htt fragments containing polyglutamine expansions aggregate faster – and their presence is more toxic to neurons –than copies of the full-length protein (reviewed in Wetzel & Mishra, 2014).

*In-vitro* biochemistry and nanoscale analysis of Htt (and other aggregation-prone proteins) using tools such as EM and atomic force microscopic (AFM) imaging are an active field and have produced many insights into kinetics and morphologies of aggregation (see e.g. Duim *et al*. 2011; Landrum & Wetzel, 2014; Legleiter *et al*. 2010; Poirier *et al*. 2002; Sahoo *et al*. 2014; Scherzinger *et al*. 1999; Thakur *et al*. 2009; Trepte *et al*. 2014). Cellular models have served as test beds to investigate the correlation between an aggregated cellular phenotype and toxicity for more than 10 years (e.g. Kar *et al*. 2014; Pieri *et al*. 2012; van Roon-Mom *et al*. 2008). So-called ‘visible aggregates’, i.e. what are herein referred to throughout as IBs, have been assayed routinely by fluorescence microscopy of puncta (typically at low magnification). These studies coarsely explore the relative prominence of aggregation *per se* in cellular populations expressing the mutant protein.

Unfortunately and often preventing further insights, optical techniques have long been limited in their ability to pick out more detailed morphologies and assess the existence of aggregated forms at intermediate stages of the process in experimental cellular systems – mainly as a result of the diffraction-limited spatial resolution available. Compared with EM or (surface-morphology-limited) AFM, the fluorescence contrast mode facilitates a much more complete appraisal – with molecular specificity – of how proteins are distributed throughout the cellular volume than could be accomplished, for example, by an immuno-gold approach. This ‘completeness’ of visualization is realized especially well when employing genetically encoded markers such as fluorescent proteins (FPs) in validated constructs for direct protein expression, since every copy of the protein of interest carries a fluorescent tag.

While fluorescence microscopy traditionally has the strong advantage of being able to image entire cells or groups of cells in real-time, also obtaining three-dimensional (3D) depth-information (e.g. by confocal sectioning), it is only through the advent of single-molecule and super-resolution microscopy methods (Moerner, 2007; Royal Swedish Academy of Sciences, 2014), based on ultrasensitive image sensor technology and instrumentation, that the full potential of the optical approach is becoming realized. Several of us (S.J.S, L.E.W., J.F. and W.E.M. with W.C. Duim) recently described technical advances which allowed the discovery of much dimmer (smaller) aggregated Htt_ex1_ species in the fluorescence mode (Sahl *et al*. 2012). Single-molecule active control microscopy (Sahl & Moerner, 2013) using the light-induced blinking (Dickson *et al*. 1997; Moerner, 1997) of enhanced yellow fluorescent protein (EYFP) (Biteen *et al*. 2008), revealed that fibrillar Htt_ex1_ aggregates can be identified in the cytosolic compartment and processes of neuronal model cells (differentiated PC12). These species, termed small aggregate species (SAS), are ~10 000–1000 000 times dimmer in the fluorescence microscope due to the much, much lower numbers of Htt_ex1_ incorporated into these fibrils compared with the large (typically ~2–8 *μ*m diameter) IBs. As a result, their visualization required us to circumvent the enormous signal discrepancies between both categories of aggregated objects by applying a dedicated protocol to photobleach or dark-state-pump the majority of fluorophores residing in the extremely bright Htt IB.

Stimulated emission depletion (STED) super-resolution microscopy (Hell & Wichmann, 1994; Klar & Hell, 1999), here utilized in the continuous-wave STED mode (Willig *et al*. 2007), readily facilitates confocal laser-scanning microscopy with focal-plane spatial resolution improved several-fold over the diffraction resolution limit (Abbe, 1873; Rayleigh, 1896; Royal Swedish Academy of Sciences, 2014). As we show below, a key strength of this approach is not just the inherent confocal optical sectioning, but the nature of point-scanning itself, which allows localized probing of fluorescence and thus inherently deals with the aforementioned issue of signal discrepancy and dynamic range (large, bright IB *versus* small, dim fibrils).

Using a combination of time-lapse and high-resolution optical approaches, we here describe the basic process of Htt_ex1_ accumulation in IBs in PC12 neuronal model cells and clarify the role of fibrils in this regard. We establish directly by imaging that inclusion body formation occurs from monomeric or low-Htt_ex1_-copy-number oligomeric protein, but does not proceed via incorporation of fibrillar precursor species. However, as aggregation progresses, an alternative – and quantitatively significant – aggregation route toward formation of cytosolic fibrillar Htt becomes available during the later stages of observation.

## Results

**Fig. 1:**
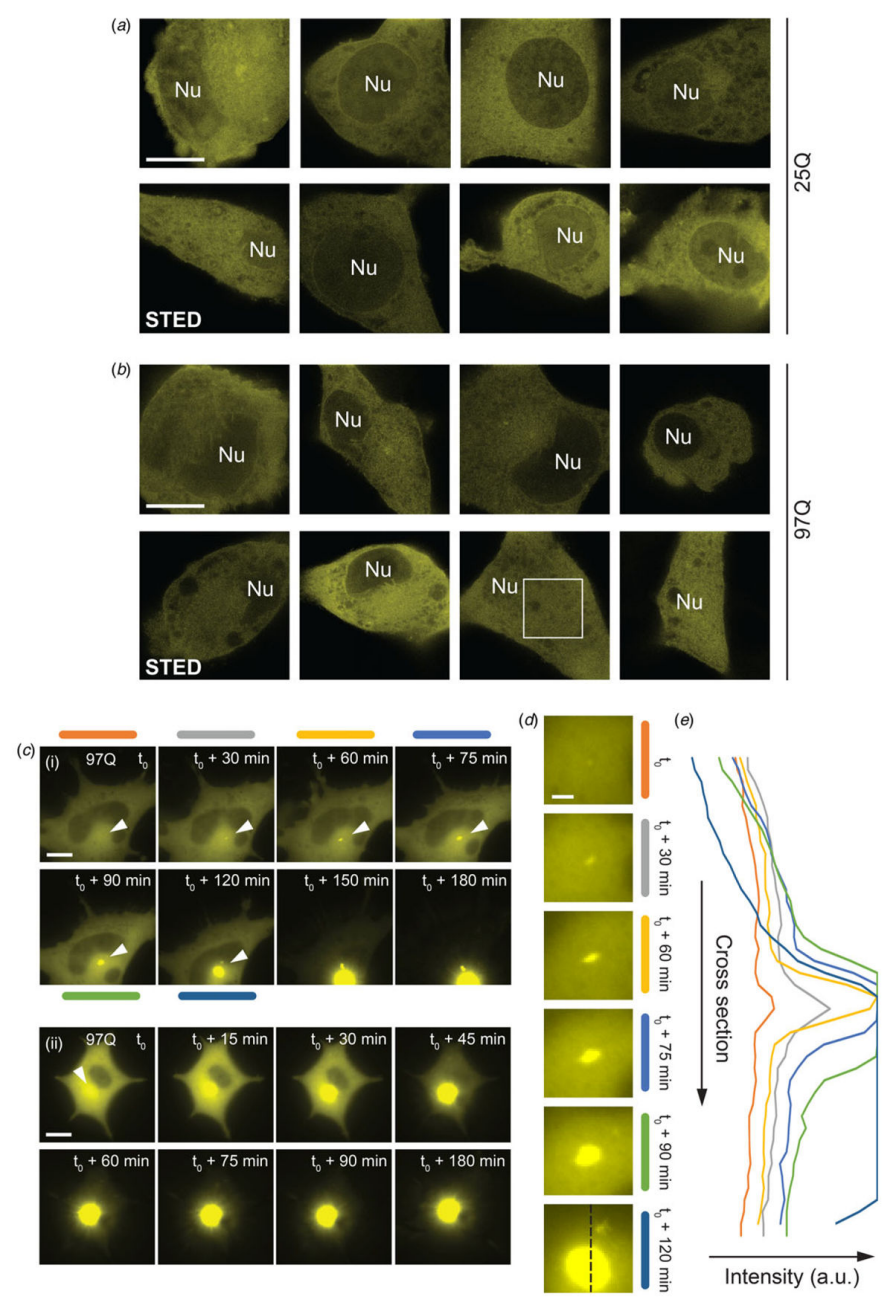
Cytosolic expression of Htt_ex1_ in NGF-treated (differentiated) PC12 cells during lag-phase (pre-aggregation), and initiation of aggregation. (*a*) Examples of cells expressing Htt_ex1(25Q)_, with uniform diffuse signal at confocal STED resolution. (*b*) Examples of cells expressing Htt_ex1(97Q)_, with uniform signal at confocal STED resolution. Nu = nucleus (inferred from other confocal section, where necessary). The images show variable but significant nuclear localization of Htt_ex1_ for both the 25Q and 97Q constructs. Signals in the white box are analyzed in Fig. 2*c*. (*c*) (i, ii) Two examples of automated time-lapse microscopy (diffraction-limited, epifluorescence), showing nucleation and growth of the inclusion body (IB), indicated by the white arrows (Htt_ex1(97Q)_). Time t_0_ indicates the time when an enhanced signal above the diffuse background can first be identified. Note that in some cases the growth of the IB is rapid and *saturates* the camera detector response (example in (ii)). Even more than ~2 h into this aggregation process, the difiuse (uniform) signal in the cell comprising monomers and possibly small oligomers exhibits no appreciable signs of additional aggregated (e.g.) fibrillar species at difiraction-limited spatial resolution. (*d*) Magnified view of nucleation and growth from example (i) (white arrows). At around 2 h, an additional dim aggregate is seen above the IB. (*e*) Vertical central fluorescence intensity cross-section as indicated at bottom of (d). Note the saturation due to the IB signal at later time points. Scale bars: 10 *μ*m (a–c), 2 *μ*m (d).

Direct cytosolic expression of fluorescent (EYFP) Htt_ex1_ constructs initially manifests as diffuse signals throughout the cellular volume in conventional microscopy, which can first be detected at moderate levels around 8–10 h after transfection of plasmid DNA. The majority of cells steadily express Htt_ex1_ without indications of aggregation. In this sub-population of cells, even 4 days after transfection, STED confocal sections at enhanced resolution do not reveal any visible clustering or aggregation for both non-pathogenic (25Q) (Fig. 1*a*) and ‘mutant’, pathogenic (97Q) (Fig. 1*b*) polyQ lengths. The 25Q constructs (Sahl *et al*. 2012) serve as comparison controls to exclude any fluorescent-tag-induced aggregation – which in our experiments is never observed – even at comparatively high expression levels (cases of brightly fluorescent cells with Htt_ex1(25Q)_). It is noteworthy with regards to the physiological relevance of the employed Htt constructs that our STED sections (~500–600 nm *z* resolution, Fig. 1*a*, *b*) directly reveal the presence of Htt_ex1_ at slightly reduced levels in the nuclear compartment, for both 25 and 97Q. Htt is known to be partially localized to the nucleus, and it is thus reassuring that our fluorescent constructs reflect this property.

Htt_ex1_ with a pathogenic polyQ tract is prone to aggregation into IBs (DiFiglia *et al*. 1997; Rajan *et al*. 2001). Diffraction-limited time-lapse microscopy (Fig. 1*c*) reveals instances of nucleation and growth of such aggregates in a perinuclear location, which quickly expand in dimension and fluorescent brightness, on the timescale of <2 h (Fig. 1*c*, (i)), and often faster (example in Fig. 1*c*, (ii), using 97Q). In the case of faster aggregation – likely as a result of more efficient transfection leading to higher mutant Htt_ex1_ concentration and/or a faster rate of mutant protein production – a matter of minutes or few tens of minutes suffices for an inclusion body to grow, whose signal quickly saturates the detector at the acquisition settings chosen to enable initial increases in signal to be (linearly) recorded (Fig. 1*d*). Figure 1*e* displays intensity cross-sections at time points up to 2 h from the instant when an enhanced localized signal can first be identified in the midst of the diffuse signal (i.e. on top of this fluorescent background).

Importantly, the time-lapse microscopy observations seem to suggest the initial growth of an IB-like aggregate in a distinct (perinuclear) location, without obvious evidence of additional small aggregates forming elsewhere in the cell at the same time or within the sub-hour time window when the growth can be mapped without detector saturation.

**Fig. 2:**
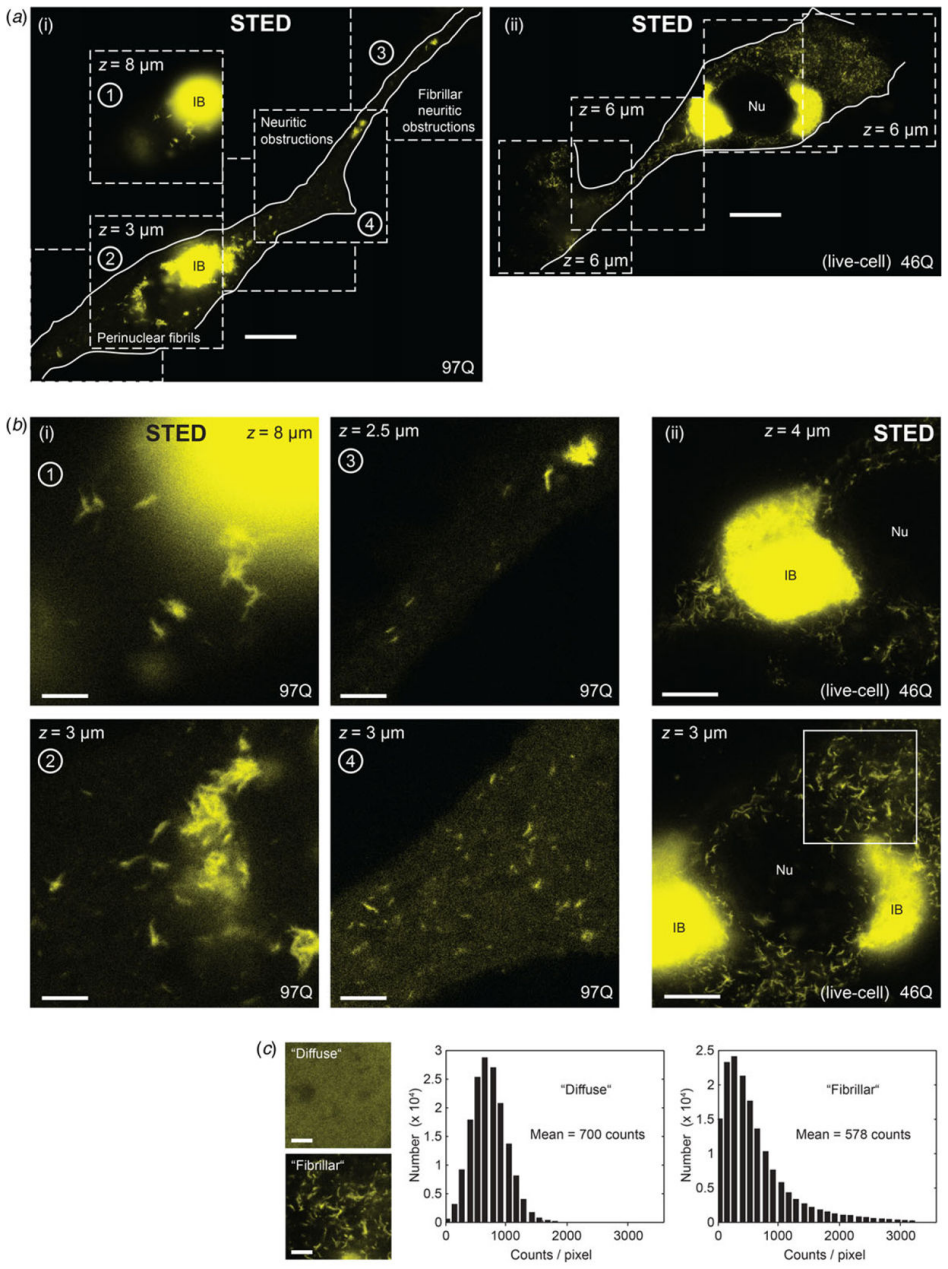
Mutant Htt_ex1_ coexists in IBs and later-stage fibrillar forms. (*a*) Cell bodies with (axonal-like) neuritic processes. Nu = nucleus, IB = inclusion body. Two examples, imaged by STED in fixed (i) and living (ii) PC12 cells (96–98 h post-transfection) are shown. (*b*) Magnified views of selected parts of the cells in (a), indicated by numbers, showing fibrils, coalescence (bundling) of fibrils in cytosol and processes. Depending on expression level, an abundance of fibrils is observed (e.g. example in (ii) at *z* = 3 *μ*m above the coverglass). Axial coordinates of STED-Confocal *z* sections are indicated. Scale bars: 10 *μ*m (a), 2 *μ*m (b,(i) and (c)) and 5 *μ*m (b,(ii)). (*c*) Histograms of pixel intensities over 10 × 10 *μ*m^2^ image fields (shown on left), comparing the non-aggregated (‘diffuse’) case (from Fig. 1*b*, white box) and aggregated fibrillar case (from Fig. 2*b*, white box).

The situation is, however, quite different in the subset of cells where aggregation into the inclusion body has already occurred. With appropriate imaging protocols (e.g. Sahl *et al*. 2012) it is possible to visualize additional, much dimmer Htt_ex1_ species. Point-scanning STED super-resolution microscopy renders the inside of mutant-Htt-expressing cells in sufficient detail to appreciate the intricate morphological characteristics of both large and small aggregated forms of the disease-causing protein (Fig. 2). Multiple confocal sections, which each discriminate against fluorescence signals from other planes, sharply outline the typically ≥2–3 *μ*m-diameter IB as a globular object of very high fluorescence intensity (Fig. 2*a*). The number of Htt copies in a mature inclusion body is estimated to be in the millions (Bates *et al*. 2015; Wetzel & Mishra, 2014). Any substructure of the inclusion body was not resolvable at the ~80 nm full width at half maximum (FWHM) (σ ≈ 35 nm) resolution level in the present experiments, but would be challenged in any case by the massive presence of (EYFP) emitters in close-by sample planes, putatively too many to reject out-of-focus fluorescence sufficiently by the confocal pinhole alone.

Cellular regions adjacent to the inclusion body, imaged at STED resolution within the same recordings, clearly reveal the presence of fibrillar Htt_ex1_ at diverse local concentrations in the cells (Fig. 2*a*, *b*). Optical super-resolution allows individual fibrils to be identified without problems, even in closely packed arrangements (examples in Fig. 2*b*). Such bundling, or ‘coalescence’, of fibrils was a feature we consistently observed when fibrils were present at higher concentration. The behavior is indeed very much akin to what has been documented for the *in-vitro* growth at higher concentration of mutant protein, also by super-resolution single-molecule fluorescence (Duim *et al*. 2011, 2014). It is worth noting that the dimmer image features – examples are seen in examples labeled 3 and 4 of Fig. 2*b* – upon closer inspection indeed reveal slightly elongated fibrillar characteristics, even the dimmest of them.

The difference between the initial diffuse cytosolic Htt_ex1_ expression (Fig. 1*a*, *b*) and the occurrence of fibrils (Fig. 2*b*) is readily apparent to the eye, and this difference is also borne out in a histogram of pixel count values over 10 × 10 *μ*m^2^ image regions for the two cases (Fig. 2*c*): While the diffuse case shows all pixels in an image section being of approximately the same signal intensity level (count) with moderate variance, the aggregated fibrillar case shows many dark pixels (positions where no Htt_ex1_ is located) and a further population of pixels of higher signal values (the tail on the histogram), where more Htt density than the diffuse level is located.

**Fig. 3:**
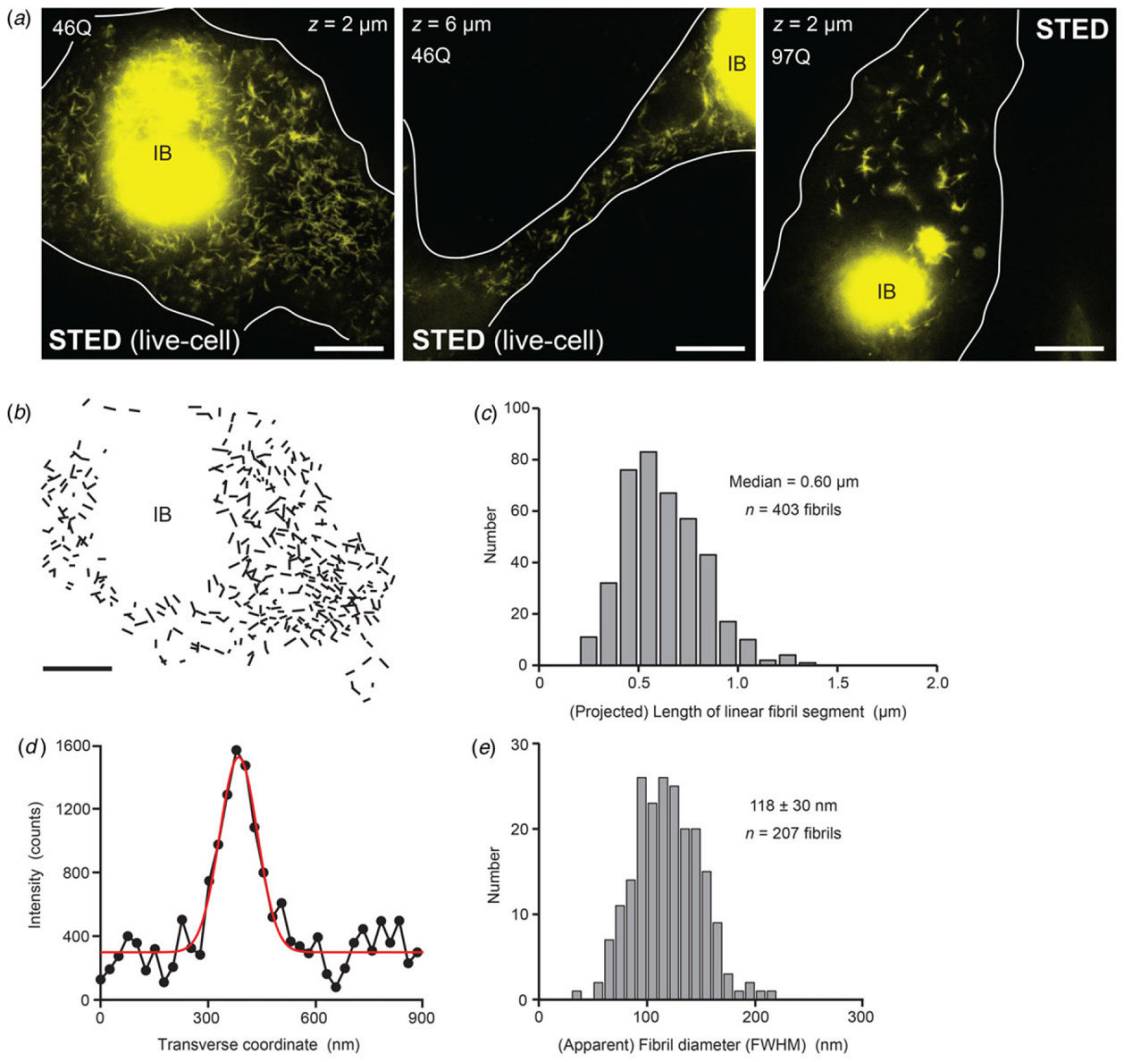
Structural characterization of fibrillar units by live-cell STED imaging. (*a*) Examples of fibrillar co-existence with inclusion bodies, imaged by live-cell super-resolution STED microscopy (first two: 98 h after transfection of plasmid DNA (middle example is an enlarged view of Fig. 2*a*, ii)) and by STED microscopy in chemically fixed cell (third example, right, at 96 h). (*b*) Outlines of identifiable linear fibril segments (from left image in (a)). (*c*) Distribution of (2D-projected) lengths of fibrillar segments (*n* = 403 fibrils measured) analyzed in (b). (*d*) Cross-sectional intensity profile across a fibril from the left image in (a). Red: Gaussian fit. (e) Width distribution of *n* = 207 individual cross-sections of fibrils (FWHM of Gaussian fit) from left image of (a).

Due to its fast sub-second acquisition of large image fields, STED imaging allowed examination of the coexistence of IBs and fibrils also in *living* PC12 cells (Fig. 2 cases (ii)). Further structural characterization of the fibrillar units in examples of image fields containing numerous fibrillar copies (Fig. 3*a*) showcases the fibril as a universal building block. Identified linear segments feature lengths of up to 1·5 *μ*m (Fig. 3*b*, *c*) and a fairly homogenous apparent width of ~120 nm, with no evidence of significant variability (Fig. 3*d*, *e*). The determined fibril widths agree well with previous determinations of ~90–110 nm (Sahl *et al*. 2012) in light of the higher spatial resolution in the previous single-molecule experiments.

The clear difference – at super-resolution – between fibrils and diffuse (i.e. *bona fide* non-aggregated) Htt is striking. It is certainly possible that the diffraction-limited level of resolution in previous studies had simply not been sufficient to reveal such signal ‘segmentation’ (compare Fig. 2*c*) at the early stage during IB formation. This stage is at a time when detection of dim species is challenged in two major ways: First, any initial concentration difference of a fibril arising amidst monomeric/oligomeric Htt is inherently challenging to detect, as the fluorescence signal *density* in the fibril must start out at the same level as its ‘diffuse’ surrounding. The second reason is the aforementioned bright IB itself, whose signal challenges the – widefield – imaging of much dimmer objects within the same field of view (Fig. 1*c*, starting at around t_0_ + 60 min, where this effect is nicely seen).

**Fig. 4:**
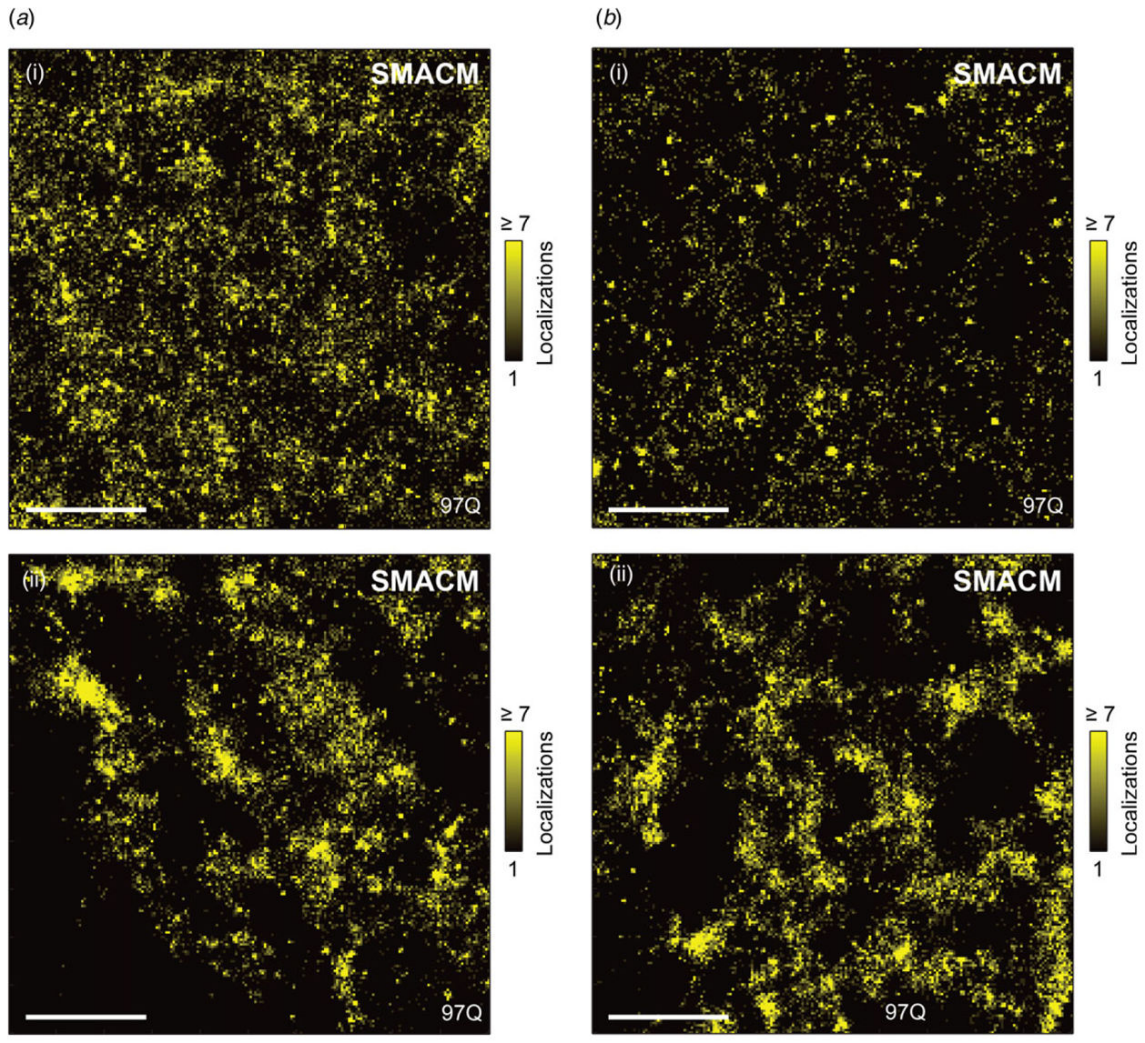
Absence of fibrillar aggregated forms during pre-aggregation lag phase and during the early inclusion body stage. (*a*) Examples of single-molecule super-resolution images of areas in cells in which no inclusion body has formed (10 h post-transfection). (*b*) Examples in a cell which contained an inclusion body elsewhere in the cell, outside the field of view shown (*t* ≈ 11 h). The bright interference of signal from the inclusion body was reduced by a targeted bleaching protocol, enabling single-molecule active control microscopy. No fibrils are seen in (*a, b*), and additional examples (all examples imaged, all are devoid of fibrillary species by inspection) are provided in a Supplementary Figure, available in high-resolution format online. Scale bars: 2 *μ*m.

This realization prompted us to perform further, more detailed super-resolution imaging using the single-molecule approach, at strategically chosen time points. We sought to accept or reject the notion that fibrils might already be present during early IB growth. To examine this, two sets of cellular samples were studied by super-resolution microscopy (Fig. 4): 1. Cells containing mutant Htt_ex1_ still in the apparently diffuse state – at the earliest possible time points (fixed at ~10 h post-transfection) when fluorescence signals first become detectable. 2. Selected cells (~10–12 h post-transfection) expressing mutant Htt_ex1_, where a bright aggregate (early, but very bright inclusion body) is already seen. For both cases, 1 and 2 (Fig. 4*a*, b), (i.e. without and with the need for the IB bleaching protocol), careful inspection of the SR reconstructions revealed the following: cells exhibited pointillist Htt_ex1_ localizations in high abundance and at variable spatial density (reflecting local expression levels and in some cases possibly enhanced local recruitments to organelles), which are clearly interpretable as individual monomers or oligomers of Htt_ex1_. Differences in the total number of localizations per spot might in principle be due to dimerization or oligomerization of Htt, but certainly have substantial contributions from the variable single-molecule photophysics of EYFP, which manifests as variable numbers of total off-on blinks and numbers of on-frames.

However, no evidence of fibrillar (oblong-shaped) objects could be obtained within this early time frame of observation (~10–12 h). Additional examples of the entirety of cells imaged (*n* = 32 and *n* = 36 separate image regions of 8 × 8 *μ*m^2^ were imaged and analyzed, respectively) are provided in a Supplementary Figure (in high-resolution).

**Fig. 5:**
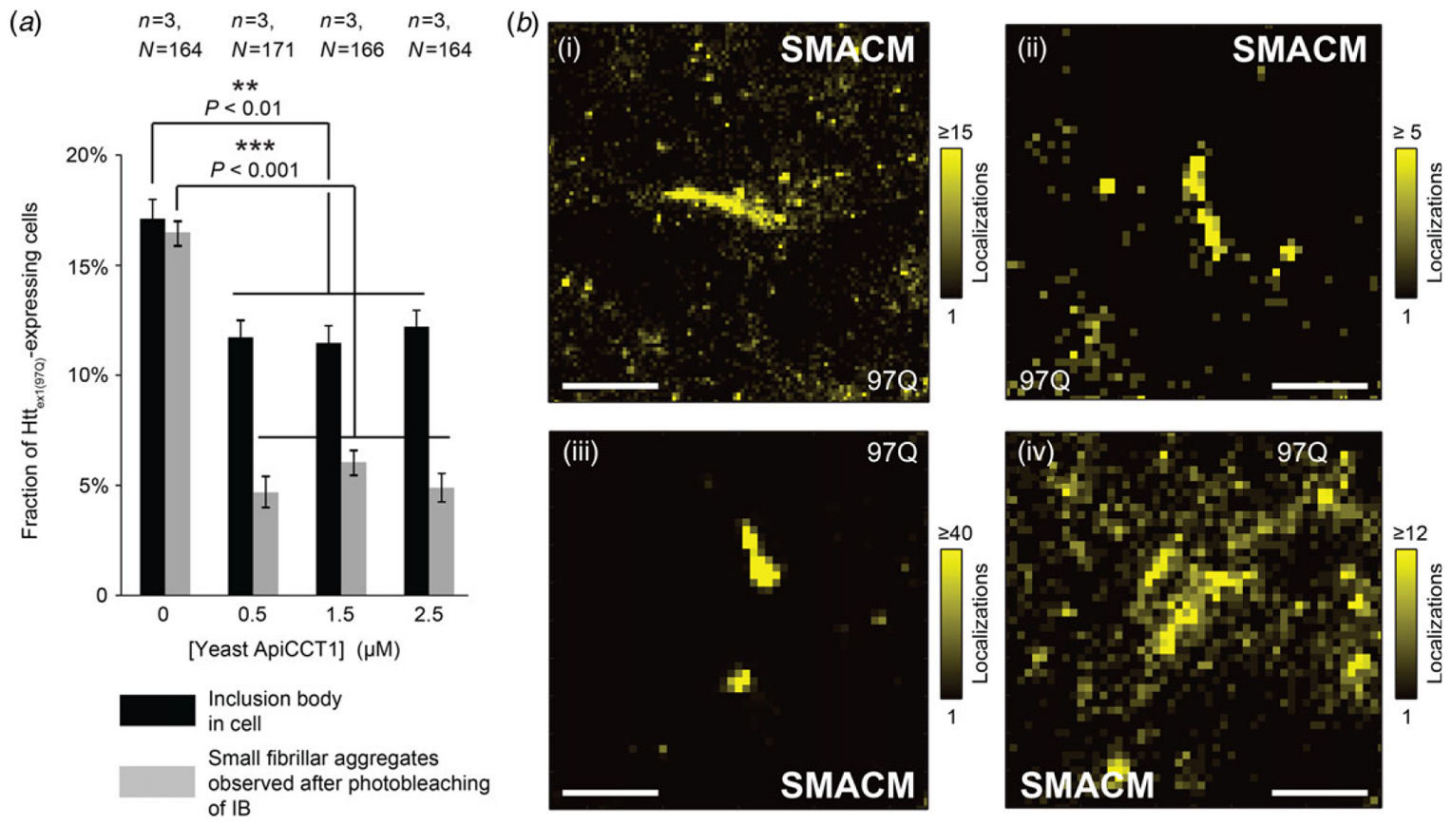
Exogenous delivery of ApiCCT1 to NGF-differentiated PC12 cells expressing mutant Htt_ex1_ substantially reduces occurrence of fibrils, more strongly than occurrence of inclusion bodies. (*a*) Fraction of transfected mutant Htt_ex1_-expressing cells containing IBs (black bars) and fibrillar SAS (grey bars) *versus* concentration of yeast ApiCCT1 supplied in cell medium for 48 h (visualization by targeted photobleaching protocol of IB). (Error bars represent mean ± S.E.M.; *n* = 3 separate groups of cells were analyzed in each case, with total numbers of cells *N* for each condition as indicated; **P < 0·01, ***P < 0·001; both one-way analysis of variance). (*b*) Examples of SAS imaged by single-molecule super-resolution microscopy (blinking of single EYFP molecules) for 1·5 *μ*M ApiCCT1, revealing fibrils of length ~300 nm to ~1 *μ*m. Scale bars: 1 *μ*m (i) and 500 nm (ii–iv).

To explore potential modulations of the formation of aggregates, previous work (Sontag *et al*. 2013) demonstrated that the cellular uptake of the apical, substrate-binding domain of the CCT1 subunit (ApiCCT1) of the CCT/TRiC chaperonin complex can be taken up by neuronal cells, where it modulates Htt aggregation. This prompted us to investigate the influence which ApiCCT1 exerts on the specific Htt_ex1_ aggregation pathways we have now partially elucidated by super-resolution microscopy, in particular, the distinct difference between IBs and fibrillar aggregates. Examination of populations of cells subjected to three concentration levels in the low *μ*M range (in cell medium) of yeast ApiCCT1 showed a clear reduction in the occurrence of intra-cellular IBs (Fig. 5*a*), in agreement with the previous report (Sontag *et al*. 2013). In that work, aggregates were referred to as ‘visible inclusions’ and their counting was conducted at low optical magnification and with much lower detection sensitivity. Application of the targeted photo-bleaching protocol for visualization of much dimmer fluorescent features allowed us to test for the presence of SAS in those cells which contained an inclusion body. While still detectable in some cases, the abundance of such SAS (here defined as dim but distinctly recognizable localized signals on the order of the diffraction resolution limit, after photo-bleaching; any presence confirmable or not) as a percentage of all mutant Htt_ex1_-expressing cells was reduced from ~16% to ~5% for ApiCCT1 concentrations of 0·5 *μ*M (and higher) (Fig. 5*a*). The ApiCCT1-mediated reduction of the fibrillar aggregate pool is therefore more pronounced than that of inclusion bodies. Importantly, the reduction of aggregation was shown to hold in the present experiments for full-exon1 variants of mutant Htt, in addition to the proline-domain-lacking variant employed by Sontag *et al*. 2013.

Single-molecule-based super-resolution microscopy revealed that the morphological characteristics (piecewise-linear fibrils) in cells treated with ApiCCT1 (Fig. 5*b*, examples shown in cells with 1·5 *μ*M ApiCCT1) may be deemed identical to those of fibrillar mutant Htt_ex1_ observed in untreated cells, further suggesting that this fibrillar growth pathway (Duim *et al*. 2014) is of a fundamental significance in cells (*in-vivo*) as well.

## Discussion

**Fig. 6:**
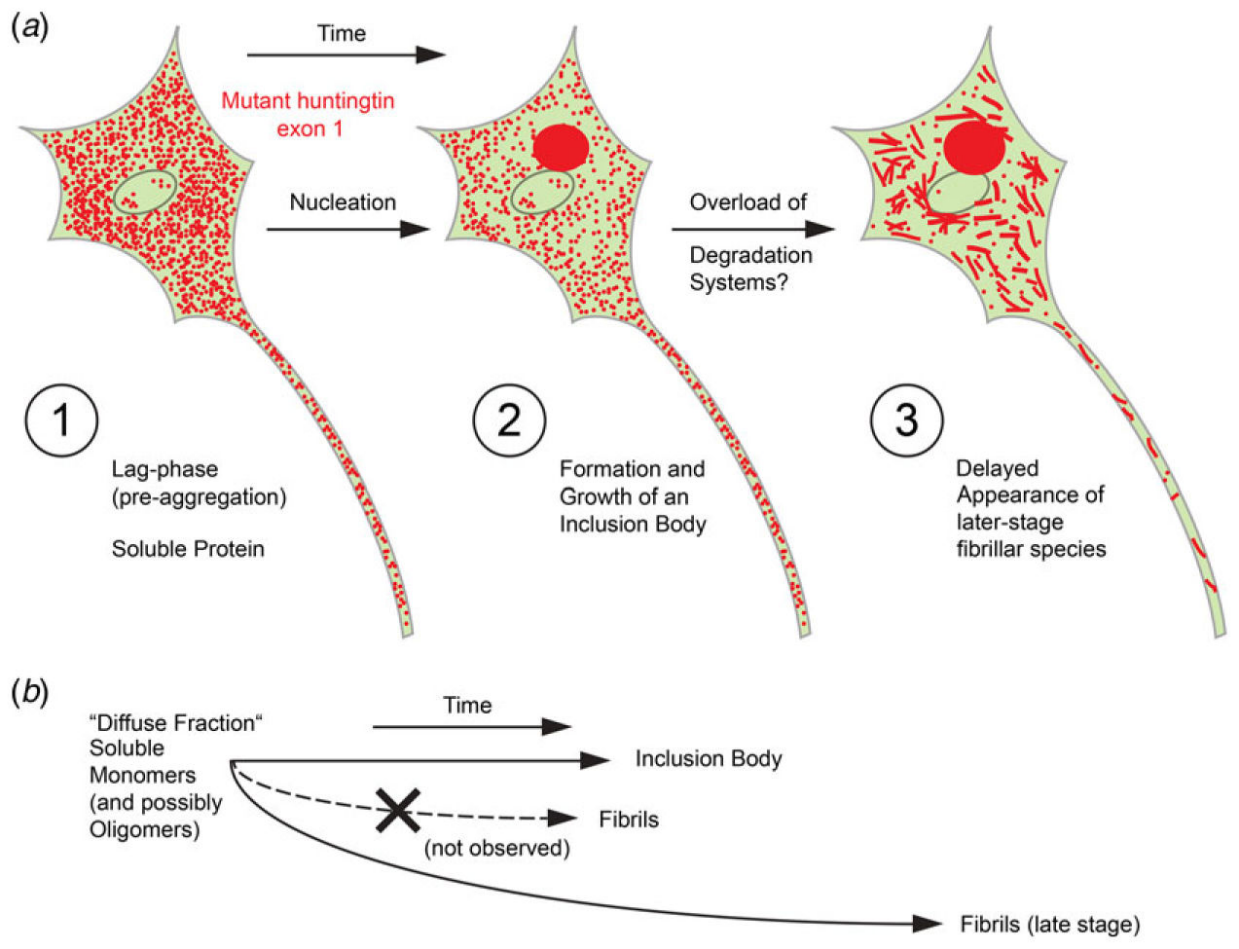
Htt_ex1_ aggregation routes suggested by super-resolution whole-cell imaging observations. (*a*) Cartoon summary of observations over time, combining insights from diffraction-limited time-lapse as well as super-resolution microscopy approaches (for details, see text). (*b*) Relationships between inclusion body and fibril formation in the cell from soluble mutant Htt_ex1_. Note that an inclusion body is formed first, and fibrils arise only at later stages, as observed in PC12 neuronal model cells.

Providing a new protein-specific window into the cell interior at enhanced spatial resolution, we have been able to visualize and untangle two populations of Htt_ex1_ aggregates and clarify the relative sequence of their genesis/formation (Fig. 6).

Our data suggest that aggregation of Htt_ex1_ into the inclusion body – which in neurons has been shown to correlate with protection from cell death (Arrasate *et al*. 2004) – may saturate the cellular degradation systems so comprehensively, to the point that cells are left vulnerable to parallel aggregation events, culminating in the emergence of additional, newly-formed fibrils (Fig. 6*a*). We find no evidence that these are present when the IB is initially forming, rather they appear to be a late-stage product. While this further aggregation route – the presence of cytosolic fibrils (Sahl *et al*. 2012) – then comes into effect, other even smaller Htt species may be yet another by-product which was difficult to disambiguate in the present experiments. In particular, amyloid fibrils with as few as <1000 protein molecules have been discussed as viable candidates for the cell-toxic species (Kar *et al*. 2014; Wetzel & Mishra, 2014), and such functional small amyloid could indeed be represented by the smallest fibrillar species which we observed (compare e.g. example 4 in Fig. 2*b* with numerous *very* dim and short fibrils).

The toxicity of fibrillar amyloid has long been discussed as a potential cause of cell death in model systems of HD (e.g. Pieri *et al*. 2012). The previous inability to comprehensively visualize Htt aggregates has been overcome by the advent of sensitive instrumentation and, in particular, the concepts for sub-diffraction-resolution (‘super-resolution’) methods (Royal Swedish Academy of Sciences, 2014).

Taken together with our previous observations (Sahl *et al*. 2012) that a fibrillar population was clearly present in the majority of IB-containing cells even more than a week after transfection (e.g. at 192 h), the data now argue for a very long-lived, persistent aggregated state (albeit the possibility of fibril disassembly has not been directly examined *in cellulo*). While much less Htt_ex1_ is contained within them than in IBs, the fibrils themselves represent over time a ‘reservoir’ of non-negligible Htt in the cytosolic compartment (Fig. 6*b*). With their high (hydrophobic) surface exposure, fibrils may pose adverse effects such as binding of other protein partners and thus interference with cellular functions.

The relative significance of the fibrillar pool may therefore be highly dependent on the exact temporal profile of mutant Htt production in the cellular space. In other words, it is likely relevant whether the maximum amount of Htt which can be sequestered in the inclusion body is reached quickly (in a situation with high expression/production of the protein). It is a hypothesis worth considering that adverse effects might only come to the fore once fibrillar species production (and possibly that of other smaller entities) is initiated.

Importantly, the experiments detailed herein do not exclude the possibility that fibrils of identical structural characteristics are the building blocks *within* inclusion bodies. This is conceivable, as direct examination of the IB formation itself at high spatial resolution has not been achieved. However, for spatially isolated fibrils to coalesce rapidly within minutes to form the origins of the IB, followed by the later attraction and incorporation of more disparate fibrils from afar is very unlikely in the crowded environment of the cell.

Our observations are more consistent with substantial growth of the IB, followed by additional ‘drawing in’ of diffuse-appearing Htt (i.e. monomers, possibly dimers or oligomers). Indeed, application of the number and brightness (N&B) approach (Dalal *et al*. 2008; Digman *et al*. 2008) previously allowed some insight into Htt_ex1_ IB formation (Ossato *et al*. 2010) in COS-7 and ST14A cells. The N&B method analyzes temporal fluorescence fluctuations within spatially diffraction-limited pixel volumes and thus cannot extract information on sub-diffraction length scales (for optical approaches, these require ‘super-resolution’ methods). Nonetheless, (Ossato *et al*. 2010) inferred a distinct step of oligomerization on the way to IBs, but obtained no evidence of brighter small aggregates such as fibrils. The regime also of interest – *after* IB formation – was described as characterized merely by depleted monomers. The fibrils are therefore novel observations, and placing them into context – also with respect to other cellular components and assessment of their specific toxicity – will likely be the subject of much research.

Some of the technical obstacles in automated time-lapse microscopy related to IB-related detector saturation could be overcome by custom-designed acquisition scripts. It would be highly informative to make dynamic observations for a longer period right during and after the onset of IB formation, especially if this can be realized at super-resolution.

Being able to image and extract structural information at the nanoscale also in *living* cells (compare Fig. 3) is a major step forward, as it excludes potential artifacts from chemical fixatives on protein nano-environments.

The ability to optically monitor the full hierarchy of aggregation-prone protein species, from the individual molecule upward, and doing so *in-situ* (*in-cellulo*), and ultimately truly *in-vivo* (compare (Berning *et al*. 2012)), at high spatial resolution, will certainly open new paths to study proteins and their aggregates in neurodegenerative diseases.

## Materials and methods

### Optical super-resolution microscopy

STED imaging at ~80 nm (FWHM, corresponding to a spatial standard deviation σ of ~35 nm) focal-plane resolution was performed on a Leica platform (TCS STED CW, Leica Microsystems). Fluorescence excitation was provided by the 514 nm line of an Argon laser, fluorescence silencing by stimulated emission depletion effected by a fiber laser at 592 nm, both in continuous-wave operation. Excitation and STED laser output powers were set to a maximum of 20% (or 5 mW) and 100% (or 1·5 W), respectively. Fluorescence was detected from 520–580 nm. STED images were typically scanned at 25·8 nm scanning steps with 8000 Hz linescans over 1024 × 1024 pixel fields. Acquisition with line averaging of 8 or 16 (for confocal) or 32 or 64 (for STED) lines produced the highest image contrast, which is beneficial especially for dim fluorescent features. No image deconvolution was applied.

Single-molecule super-resolution imaging was performed on a custom optical setup built around an inverted microscope (Olympus IX-71) with motorized stage (ProScan III, Prior) and the sample mounted in a high-stability custom holder (Tokai Hit). The excitation/photoblinking light (514 nm, Coherent Sapphire) was focused into the back aperture of a 1·4 NA oil-immersion objective (Olympus UPLSAPO, 100X), achieving standard Köhler illumination for wide-field laser excitation (~20–30 *μ*m spot size) in epifluorescence mode. Fluorescence was selected by a dichroic mirror (Semrock, Di01-R532), a 525 nm long pass filter (Semrock, BLP01–514R-25) and a band pass filter for EYFP emission (Semrock, FF01–578/105–25). The image was formed on a highly sensitive electron-multiplying charge coupled device (Si EMCCD) camera (Andor iXon897 Ultra) acquiring at 35 ms per frame. Single molecules of EYFP blinked vigorously on and off under ~2–3 kW cm^−2^ illumination with 514 nm, and localizations could be extracted using the program RapidSTORM (Wolter *et al*. 2010) (which had been independently validated on other test structures under similar signal-to-background single-molecule conditions). Reconstructed super-resolution images (from localizations with on average ~1500–2000 photons per event, localization precision σ of ~15–20 nm) were computed as histograms, i.e. numbers of localizations falling within 40 × 40 nm pixel domains.

### Video microscopy

Time-lapse (diffraction-limited) imaging was performed on a Zeiss Axio Observer. Z1 epifluorescence microscope at physiological temperature, using automated acquisition of collections of cells.

### Htt_ex1_-expressing PC12 neuronal model cells

Fluorescent Htt constructs (EYFP, fused to C terminus of complete exon 1 with indicated number of Q repeats) were expressed by standard transfection protocols as described previously (Sahl *et al*. 2012). The key points are summarized again here.

### Fluorescent Htt_ex1_

The entire exon 1 of Htt (Htt_ex1_) MATLEKLMKAFESLKSF [nQ]PPPPPPPPPPPQLPQPPPQAQPLLPQPQPPPPPPPPPPGPAVAEEPLHRPGS) with an nQ polyglutamine tract was cloned into a mammalian expression vector (pcDNA3·1(−), Invitrogen) using XhoI and BamHI restriction enzymes (New England Biolabs) resulting in CMV-promoter-controlled (CMV: cytomegalovirus) C-terminal fusion constructs of Htt_ex1_ to EYFP. Our studies used *n* = 25 as a non-pathogenic control, with *n* = 46 and 97 leading to aggregation.

### Cell culture

PC12 cells were grown to confluence at 37 °C in a 5% CO_2_, 96% relative humidity incubator in tissue-culture treated flasks (75 cm^2^, BD Biosciences) in cell culture medium (10% fetal bovine serum (FBS), 90% phenol red free DMEM, both Gibco). Cells were passaged with 1:5 dilution at least 3 times prior to experiments. Each passage consisted of 5 min incubation with 2 ml of trypsin replacement (TrypLE Express, Gibco), addition of 8 ml cell media to deactivate the enzyme, a brief centrifugation, followed by media replacement to remove any residual trypsin replacement. Cell suspensions were then added to 6-well tissue culture plates (BD Biosciences). Cells grew to a monolayer (>90% confluency in 1–2 days), whereupon differentiation into sympathetic-neuron-like cells was achieved by treatment with nerve growth factor (NGF 2·5S, ~100 ng ml^−1^, Invitrogen) for 48 h. At this time, cells were transfected with the plasmid DNA for fluorescently labeled Htt_ex1_ utilizing a modified protocol for Lipofectamine 2000 (Invitrogen).

### Transfection

Complexes were prepared using 2 *μ*g of DNA and 6 *μ*l of Lipofectamine 2000 in 100 μl of Opti-MEM 1 media (Gibco). Complexes of DNA with transfection reagent were then added to cells and incubated for 5 h, at which point the transfection-complex-containing medium was aspirated and each well was treated with 0·5 ml TrypLE Express for 5 min. Cells were suspended in 4–8 ml of NGF-supplemented media and were divided between 2–4 wells (to achieve appropriate cell densities for imaging) in 6-well plates containing microscope slides. These slides (standard no. 1 coverslips, Fisher) were pre-cleaned, ozone-treated and coated with 500 *μ*l of 8·4 *μ*g ml^−1^ fibronectin (EMD Biosciences) in 1× PBS (pH 7·4, Gibco), and rinsed with 1× PBS before use. At defined time points post-transfection, cells were imaged alive, or fixed in 4% paraformaldehyde (Electron Microscopy Sciences) and washed for the extended, detailed cell-by-cell examination and super-resolution imaging.

### Medium (exogenous) delivery of chaperonin subunit fragment ApiCCT1

Following a previous protocol (Sontag *et al*. 2013), the 6×-his-tagged (yeast) recombinant ApiCCT1 (MVPGYALNCTVASQAMPKRIAGGNVKIACLDLNLQKARMAMGVQINIDDPEQLEQIRKREAGIVLERVKKIIDAGAQVVLTTKGIDDLCLKEFVEAKIMGVRRCKKEDLRRIARATGATLVSSMSNLEGEETFESSYLGLCDEVVQAKFSDDECILIKGTSKAAAAALEHHHHHH) was added to the cell medium 8 h after transfection of the Htt constructs at the concentration stated during growth for a total of 48 h before chemical fixation (as above) and examination by microscopy.

## Supplementary Material

1
