# Supplementary material for: Delayed emergence of subdiffractionsized mutant huntingtin fibrils following inclusion body formation
Source: Q Rev Biophys. Author manuscript; Available in PMC 2017 Jan 1. (PMC4785097; doi:10.1017/S0033583515000219)
Supplement: 1 [file NIHMS740886-supplement-1.pdf]

Delayed emergence of subdiffraction-sized mutant huntingtin fibrils following inclusion body formation

Steffen J. Sahl[1,┼*], Lana Lau[1], Willianne I. M. Vonk[2], Lucien E. Weiss[1], Judith Frydman[2] & W. E. Moerner[1*]

Departments of [1]Chemistry and [2]Biology, Stanford University, Stanford, CA, USA

┼Present address: Max Planck Institute for Biophysical Chemistry, Göttingen, Germany

Correspondence should be addressed to W.E.M. (wmoerner@stanford.edu) or S.J.S. (steffen.sahl@mpibpc.mpg.de).

Supplementary Figure | Additional examples of single-molecule super-resolution imaging.
(**a**) Cells containing mutant Htt_{ex1} (97Q) still in the apparently diffuse state – at the earliest possible time points (fixed at ~10 hours post-transfection) when fluorescence signals first become detectable. (**b**) Selected cells (~10-12 hours post-transfection) expressing mutant Htt_{ex1} (97Q), where a bright aggregate (early, but very bright inclusion body) is already observed. The regions imaged do not contain the inclusion body, which has been dramatically reduced in fluorescence intensity by a targeted bleaching protocol. In both cases, regions of 8×8 μm^2 were imaged. Scale bars: 2 µm.

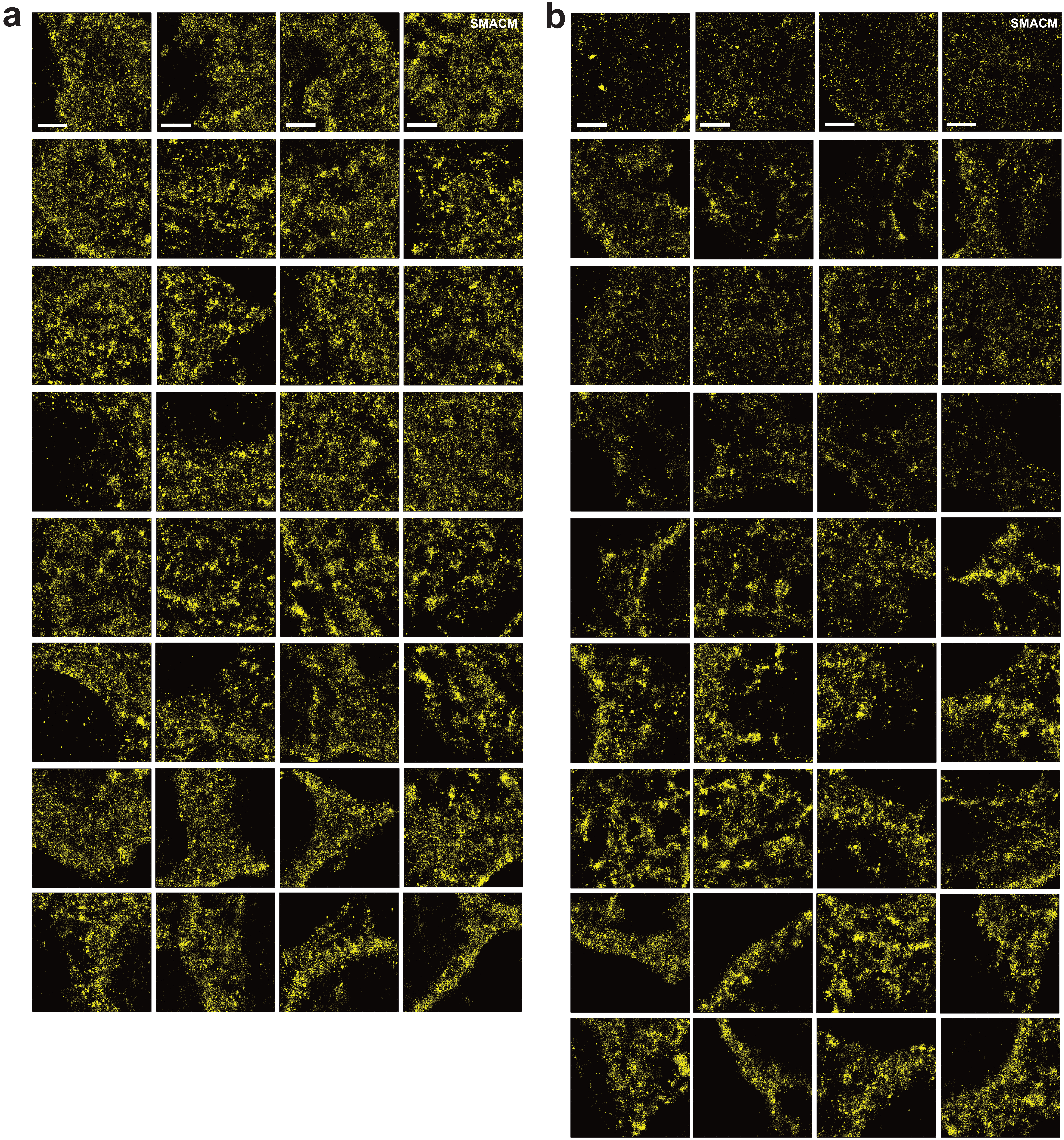
a
SMACM
b
SMACM
